# β-MSCs: successful fusion of MSCs with β-cells results in a β-cell like phenotype

**DOI:** 10.18632/oncotarget.10214

**Published:** 2016-06-21

**Authors:** Zahra Azizi, Claudia Lange, Federico Paroni, Amin Ardestani, Anke Meyer, Yonghua Wu, Axel R. Zander, Christof Westenfelder, Kathrin Maedler

**Affiliations:** ^1^ Centre for Biomolecular Interactions, University of Bremen, Bremen, Germany; ^2^ Department of Cell and Gene Therapy, University Medical Center Hamburg-Eppendorf, Hamburg, Germany; ^3^ Departments of Medicine and Physiology, University of Utah, Salt Lake City, Utah, USA; ^4^ George E Wahlen Department of Veterans Affairs Medical Center, Salt Lake City, Utah, USA; ^5^ German Center for Diabetes Research (DZD) Project Partner, University of Bremen, Bremen, Germany; ^6^ Department of Laboratory Medicine, Peking University Third Hospital, Beijing, China

**Keywords:** MSCs, cell fusion, beta-cells, islets, diabetes, Pathology Section

## Abstract

Bone marrow mesenchymal stromal cells (MSC) have anti-inflammatory, anti-apoptotic and immunosuppressive properties and are a potent source for cell therapy. Cell fusion has been proposed for rapid generation of functional new reprogrammed cells. In this study, we aimed to establish a fusion protocol of bone marrow−derived human MSCs with the rat beta-cell line (INS-1E) as well as human isolated pancreatic islets in order to generate insulin producing beta-MSCs as a cell-based treatment for diabetes.

Human eGFP^+^ puromycin^+^ MSCs were co-cultured with either stably mCherry-expressing rat INS-1E cells or human dispersed islet cells and treated with phytohemagglutinin (PHA-P) and polyethylene glycol (PEG) to induce fusion. MSCs and fused cells were selected by puromycin treatment.

With an improved fusion protocol, 29.8 ± 2.9% of all MSCs were β-MSC heterokaryons based on double positivity for mCherry and eGFP.

After fusion and puromycin selection, human *NKX6.1* and *insulin* as well as rat *Neurod1, Nkx2.2, MafA, Pdx1* and *Ins1* mRNA were highly elevated in fused human MSC/INS-1E cells, compared to the mixed control population. Such induction of beta-cell markers was confirmed in fused human MSC/human dispersed islet cells, which showed elevated *NEUROD1, NKX2.2, MAFA, PDX1* and *insulin* mRNA compared to the mixed control. Fused cells had higher insulin content and improved insulin secretion compared to the mixed control and insulin positive beta-MSCs also expressed nuclear PDX1. We established a protocol for fusion of human MSCs and beta cells, which resulted in a beta cell like phenotype. This could be a novel tool for cell-based therapies of diabetes.

## INTRODUCTION

Diabetes has become a worldwide health problem in our society and curative therapies to restore the insulin producing beta cells are urgently needed. Destruction and failure of pancreatic beta cells to produce sufficient amounts of insulin to maintain normoglycemia are the main reasons for type 1 diabetes (T1D) as well as type 2 diabetes (T2D). Islet transplantation together with an improved immunosuppressive therapy [1] is one source for new beta cells and a way to restore euglycemia in patients and evades the essential need for exogenous insulin injection, although only for a limited time because of the decline in islet survival with time. Donor islet cells are limited and insufficiently available for diabetes therapy and the necessary immunosuppression is often too risky to justify transplantation in patients with long standing T1D, where infections often occur with increased severity [2].

Current studies show that co-transplantation of MSCs with pancreatic islets prolongs islet survival after transplantation due to the unique hypo-immunogenic, immunomodulatory, and anti-apoptotic effects of MSCs [3-5]. MSCs differentiate into mesodermal lineages like osteocytes, chondrocytes, adipocytes, and tenocytes *in vitro* [6] and do not form teratomas *in vivo* [7]. They have been recently suggested as a potential cellular source for regenerative therapy also for diabetes with various mechanisms to support β-cell protection [8, 9]. On the other hand, their immunomodulatory effect through paracrine factors and no transdifferentiation capacity has been defined in several studies to be the main mode of action [4, 10-12].

MSCs are identified by their cell membrane markers (CD105^+^, CD90^+^, CD73^+^) and by the lack of hematopoietic surface markers and those, which activate the host immune system (HLA-DR^−^, CD14^−^, CD80^−^, CD86^−^, CD45^−^, CD34^−^, CD79^−^) [13]. They are easy to isolate from the bone marrow and rapidly expandable *in vitro*. After transplantation, MSCs act as suppressors of immune responses by producing anti-inflammatory cytokines and growth factors, which inhibit monocyte maturation and T-/B-cell proliferation but also modulate mitogenesis, apoptosis and cell growth [14-17]. The immunomodulatory effect was shown in MSC/islet co-transplantation increasing the number of regulatory T cells (Tregs) in rodents and nonhuman primates [3, 18]. Induction of insulin-producing cells out of MSC without gene transfer was observed *in vitro* leading to reduced blood glucose levels after transplantation [19].

Co-transplantation of MSCs together with islets into diabetic mouse models successfully improved islet function and graft survival as well as glycemia [4, 18, 20-24], which were induced by MSC-enhanced tissue repair and improved re-vascularization. MSCs also improved β-cell survival, insulin secretion and insulin sensitivity in a T2D model, mainly through their paracrine effects [25]. Together, these studies show the potential of MSCs for β-cell repair in the pancreas for diabetes therapy. There is still the open question of a possible advantage of β-cell fusion with MSCs.

Cell-cell fusion, when two cells are fused into one, initiates a rapid differentiation process [26]. This phenomenon naturally occurs during development, e.g. the formation of polyploid muscle (myocyte) or bone (osteoclast) cells [27, 28], or in adult tissue repair as well as in immune response [29]. The fusion event can be induced through three different methods; physically (electric pulses), chemically (polyethylene glycol; PEG) with random pairing and low efficiency or biologically (inactivated virus) [30-32]. Cell fusion results in three distinct outcomes; heterokaryon or homokaryon, synkaryon and hybrid cells. Heterokaryons are polyploid non-dividing cell and often in a transient state, their nuclei will fuse later resulting in a polyploid synkaryon in which a cell has a nucleus with a combined chromosome pool of all nuclei. Proliferating synkaryons make hybrids. Heterokaryons offer a unique opportunity to trace the variation of chromosome pools in an intact nucleus after the fusion event [33].

During cell fusion, epigenetic and genetic information of different cell types are combined. When two distinct types of cells fuse, the encoding of a group of genes activates resulting in a modified cellular expression pattern. This event starts within a few hours in the heterokaryon state by remodeling chromatin and switching on trans-acting regulators at key loci [26, 34, 35].

The proof of concept that successful cell fusion can lead to stable functional β-cell like cells has been established previously [36, 37]. By electrofusion of immortal human PANC-1 epithelial cells with human pancreatic islets McCluskey et al. established a functional human beta cell line (1.1B4) [36]. Yanai et al obtained stable functional cells by electrofusion of rodent MSCs and islet cells [37]. Both studies show robust functional fused cells with beta cell marker expression. Importantly, the fused cells lead to a reduction in glucose levels when transplanted into STZ–diabetic mice. In concordance with such previous work, our study also shows functional fused cells of human origin upon chemical fusion. By combining the multipotent, anti-apoptotic, immunogenic and tissue repair capacity of the MSCs with the beta cell specific insulin production, we aimed to establish a stable novel beta cell type. Here we describe an optimized virus-free cell fusion protocol and produced β-MSC heterokaryons by fusion of human MSCs with rat INS-1E cells or with dispersed human islet cells to generate differentiated β-MSCs.

## RESULTS

### Generation of rat-human β-MSC heterokaryon cells by PEG-mediated cell fusion

**Figure 1:**
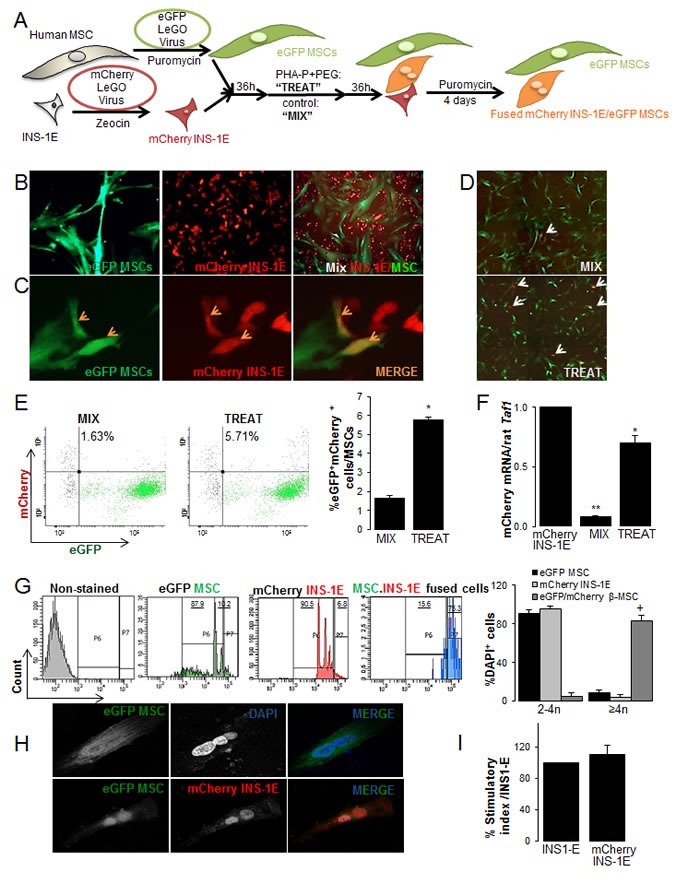
Generation of rat-human β-MSC heterokaryons (**A**) Schematic illustration of the strategy for generation of human stable eGFP^+^MSCs and rat mCherry^+^INS-1E cells that carry antibiotic-selectable markers. Cells were mixed (“MIX”) or fusion was induced by treatment with 100 μg/ml PHA-P and 50%W/V PEG (“TREAT”). Interspecies heterokaryons and human MSCs were selected by puromycin for 4 days. (**B**) Cultured eGFP^+^MSCs (left; 200X), mCherry^+^INS-1E (middle; 200X) and MIX (right: 100X) and (**C**) mCherry^+^INS-1E, eGFP^+^MSC and double positive β-MSC (orange arrows) in TREAT condition (400X). (D-H) TREAT cells after puromycin selection. (**D**) MIX (control) and TREAT conditions after 4 days puromycin selection, white arrows show eGFP^+^mCherry^+^ cells (40X). (**E**) Analysis and quantification (right) of mCherry^+^eGFP^+^ cells in MIX and TREAT conditions by FACS analysis. Gating was set up with single mCherry or single eGFP+ cells and conditions kept constant. (**F**) The relative mCherry mRNA expression normalized to INS-1E cells. RT-PCR was normalized to rat *Taf1* (**G**) Polyploidy analysis by FACS of eGFP^+^MSCs, mCherry^+^INS-1E (controls) and eGFP^+^mCherry^+^ heterokaryons. Gating was set for control non-stained cells and kept constant. (**H**) An eGFP^+^ polyploid cell (upper panel) und an mCherry^+^eGFP^+^ polyploid heterokaryon from the eGFP^+^MSCs- mCherry^+^INS-1E fusion by CLSM (400X). (I) Insulin secretion of mCherry INS1-E in compare to INS-1E cells by glucose stimulation. All analyses were performed in at least three independent experiments from three MSC donors and show mean ± SEM. *P< 0.05 Treat compared to Mix. ** Mix compared to mCherry INS-1E alone. +P< 0.05 eGFP^+^mCherry^+^β-MSC compared to eGFP^+^MSC or to mCherry^+^INS-1E.

In this study, cell-cell fusion was established with the aim to reprogram MSCs to β-cells. MSCs from human bone marrow showed CFU-F activity based on crystal violet staining (Suppl. Figure 1A), expressed cluster of differentiation CD105, CD90 and CD73, lacked expression of CD45, CD34 and MHC-II (Suppl. Figure 1A, 1B) and could differentiate into osteoblasts and adipocytes (Suppl. Figure 1C). To identify and select cells, we infected human MSCs with eGFP-puromycin and INS-1E cells with mCherry-zeocin lentiviral gene ontology (LeGO) viruses and made stably eGFP^+^MSCs by puromycin and mCherry^+^INS-1E by zeocin mediated antibiotic selection. eGFP^+^ or mCherry^+^ cells were detected by flow cytometry at 3 weeks after antibiotic selection. Microscopic and flow cytometry analyses both showed that cells were eGFP labeled MSCs or mCherry labeled INS-1E (Figure 1B, Suppl. Figure 1D). EGFP^+^MSCs stained positive for CD105, CD73 and CD90 (Suppl. Figure 1E) and mCherry^+^INS-1E for insulin (Suppl. Figure 1F). Based on the glucose stimulation insulin secretion assay, functionality of mCherry-INS1-E was similar to non-transfected INS1-E cells (Figure 1I). To generate interspecies heterokaryons, we established an optimized cell fusion protocol of MSCs isolated from human bone marrow and the rat INS-1E beta cell line (Figure 1A). We co-cultured (“MIX”) eGFP^+^ human MSCs and mCherry^+^ rat INS-1E cells in INS-1E (RPMI+FCS) or in MSC medium (AlphaMEM+ platelet lysate: PL) and selected INS-1E for all further experiments (Suppl. Figure 1G). After 12 and 36 hours, adherent co-cultured cells were induced (”TREAT”) with 100 μg/ml PHA-P for 30 min and 50% W/V PEG for 40 seconds to promote fusion, which resulted in three cell populations; eGFP^+^MSCs, mCherry^+^INS-1E and double positive heterokaryons (Figure 1C).

**Figure 2:**
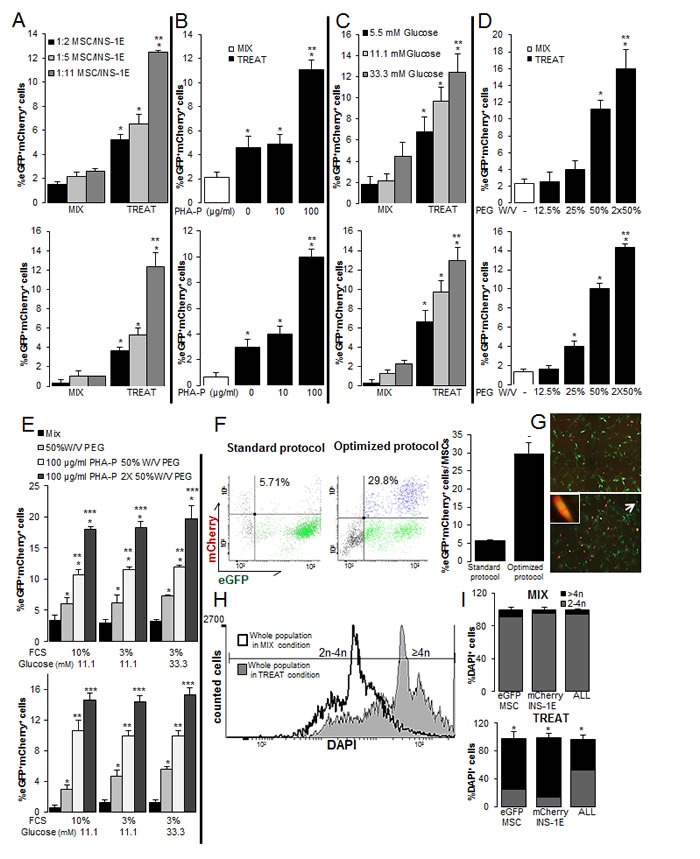
Optimization of the PEG-mediated cell fusion for rat-human β-MSC heterokaryons (**A**-**E**) The percentages of MSC/INS-1E cells measured by eGFP^+^mCherry^+^ flow cytometry (top) as well as quantitative fluorescent microscopic analysis (bottom) in MIX and TREAT conditions after puromycin selection of all cells in culture. (**A**) The mixture of the 1:2 ratio of MSC/INS-1E cells (standard protocol), 1:5 or 1:11 in mixed cells (MIX) and 50%W/V PEG and 100 μg/ml PHA-P treated cells at 11.1 mM glucose as the control glucose concentration for INS-1E cells. (B-E) MSC/INS-1E cells at a ratio of 1:11. (**B**) 50%W/V PEG treated cells were exposed to 0, 10 and 100 μg/ml PHA-P. (**C**) 50%W/V PEG and 100 μg/ml PHA-P treated and untreated mixed cells were pre-incubated for 8 h with 5.5, 11.1 or 33.3 mM glucose. (**D**) 100 μg/ml PHA-P treated were exposed to 12.5, 25 and 50% W/V or twice 50% W/V PEG. (**E**) 1X or 2X 50%W/V PEG and 100 μg/ml PHA-P treated cells and mixed cells were pre-incubated with media including 10% FCS, 3% FCS or 3% FCS/ 33.3 mM glucose. (**F**, **G**) Comparison of the number of eGFP^+^mCherry^+^ MSCs to all MSCs between standard and optimized protocol (1:11 MSC/INS-1E; 2×50%W/V PEG and 100 μg/ml PHA-P) by flow cytometry analysis of mCherry and eGFP (**F**) and by fluorescence microscopy (G; 40X; right). Arrow bar shows an eGFP^+^mCherry^+^ MSC in higher magnification (400X). The difference in A-E and F is in the normalization strategy; while results in A-E are normalized to all cells in culture, in F they were normalized to all MSCs. (H, I) Polyploidy analysis by FACS before puromycin selection of mixed and treated cells (**H**) and quantification of polyploid cells within eGFP^+^MSCs or mCherry^+^INS-1E cells alone and the whole population (eGFP^+^MSCs/mCherry^+^INS-1E/eGFP^+^mCherry^+^β-MSC) (**I**). Average counted cells were 5000 events per condition. Analyses were performed in at least three independent experiments and show mean ± SEM. *P< 0.05 Treat compared to Mix at the same conditions; **P< 0.05 at the ratio of 1:11 compared to 1:5 (A), 100 compared to 10 μg/ml PHA-P (B), 33.3 compared to 5.5 mM glucose (C), 2X compared to 1X 50% W/V PEG (D), PHA-P/PEG compared to PEG alone, ***P<0.05 2x compared to 1x 50% W/V PEG at the same conditions, ^−^P< 0.05 optimized compared to standard protocol;

Another 36 hours later, puromycin was added to treated or mixed cells for 4 days (Suppl. Figure 1G) in order to select MSCs and β-MSC fused cells. This resulted in the two main populations; eGFP^+^MSC and fused eGFP^+^MSC/mCherry^+^INS-1E cells. Again, double eGFP^+^/mCherry^+^ cells were seen in the treated cells, but only a few double positive cells were observed in the mixed control cells (Figure 1D). This was confirmed by double positive FACS analysis for eGFP/mCherry, which showed 1.6% spontaneous fusion in MIX control and this percentage increased 3-fold in TREAT conditions (Figures 1E, 2A). The level of mCherry expression was significantly higher in TREAT compared to MIX (Figure 1F). When the same experiment was performed in suspension culture, cells only had a limited capacity to attach to the culture dish and to survive after treatment (not shown).

To investigate whether the generated fused cells are polyploid heterokaryons and to confirm the efficiency of fusion, cells were fixed and labeled with DAPI. In contrast to single eGFP^+^MSCs or mCherry^+^INS-1E cells, where the majority of cells were diploid, 83±5.8% of the treated eGFP/mCherry double positive cells were polyploid based on FACS analysis (Figure 1G). Polyploidy as well as eGFP/mCherry double positivity of cells in culture was further confirmed by confocal microscopy (Figure 1H, Suppl. Figure 1H).

### Optimization of the PEG-mediated cell fusion for rat-human β-MSC heterokaryons

Because of the known important effects of the ratio of nuclear and cytoplasmic factors [26], we hypothesized that a higher ratio of INS-1E to MSCs may increase the number of puromycin selected fused heterokaryons. Therefore, we co-cultured MSC/INS-1E cells at an increasing ratio (1:2; 1:5 and 1:11) at the control 11.1 mM glucose concentration for INS-1E cells. This strategy resulted in a 2-fold further increased number of fused cells at a ratio of 1:11 compared to 1:2, based on eGFP/mCherry double positive cells analyzed by FACS analysis (Figure 2A upper panel, Suppl. Figure 2A), which was confirmed by counting the double positive cells under the microscope (3-fold increase; Figure 2A lower panel). In the next step, we further optimized the fusion protocol at the 1:11 MSC/INS-1E ratio by applying different concentrations of PHA-P, PEG and glucose. 30-min pretreatment of 10 μg/ml PHA-P had no effect, but 100 μg/ml PHA-P resulted in 3-fold increase in eGFP/mCherry double positive cells, compared to control without PHA-P, analyzed by FACS (Figure 2B upper panel, Suppl. Figure 2B), and microscopical analysis (Figure 2B lower panel). Glucose is the major fuel for the β-cell and elevated glucose concentrations have a dual function, proliferation in the short- apoptosis and functional damage (glucotoxicity) in the long term. Therefore, we hypothesized that stimulating the β-cells with 33.3 mM glucose may increase the number of β-MSCs. The number of eGFP/mCherry double positive cells was further increased by 8-hour pre-treatment with elevated 33.3 mM glucose concentrations compared to 5.5 mM glucose (Figure 2C, Suppl. Figure 2C). Increasing the concentration of PEG to 50% W/V induced a 4-fold increase in heterokaryons, compared to 12.5% PEG (Figure 2D, Suppl. Figure 2D) and two times 50% W/V PEG treatment further increased the number of double positive cells significantly (Figure 2D, Suppl. Figure 2D).

Since starvation is another stress condition, we chose mild cell starvation before fusion and reduced the FCS concentration from 10% to 3% for 8 hours. This resulted in no change of eGFP/mCherry heterokaryons at 3% compared to 10% FCS pre-culture, nor at 3% FCS under 33.3 mM glucose. But we constantly confirmed the effect of PHA-P as well as 1X and 2XPEG treatment in all conditions, which constantly showed a higher percentage of eGFP/mCherry double positive cells analyzed by FACS analysis (Figure 2E upper panel, Suppl. Figure 2E) and by counting (Figure 2E lower panel), compared to the mix condition. Microscopic and flow cytometry analyses both showed more eGFP+ mCherry + cells in “optimized” fusion protocol (MSC/INS-1E cells ratio of 1:11, 100 μg/ml PHA-P, 2X 50% W/V PEG) compared to the first “standard” fusion protocol (MSC/INS-1E cells ratio of 1:2, 100 μg/ml PHA-P, 50% W/V PEG; Figure 2F) (Figure 2F, 2G). FACS analysis indicated 29.8 ± 2.9% eGFP/mCherry co-positivity of all MSCs in culture in our optimized protocol, which are 5-fold more heterokaryons, compared to the first “standard” fusion protocol.

Our optimized condition resulted in a shift from diploid state (2-4n) in mixed control cells to polyploid state (≥4n) in treated cells (histogram, Figure 2H, Suppl. Figure 2F). In order to investigate all cells, we did not select the cells by puromycin. While almost all untreated cells were diploid, 72.8 ± 9.7% of MSCs, and 85.6 ± 6.5% of INS-1E cells and 44.1 ± 6.0% of the whole cell population (eGFP^+^MSCs/mCherry^+^INS-1E/eGFP^+^-mCherry^+^β-MSC) were polyploid after treatment (Figure 2I). In order to monitor the effect of PHA-P/PEG on cell survival, viability was analyzed before selection. The number of cells in culture, cell viability measured by tryptophan (Suppl. Figure 2G) as well as their protein content (Suppl. Figure 2H) did not change in the fused compared to the MIX conditions after PHA-P/ PEG treatment at the optimized conditions of 100 μg/ml PHA-P, 2X 50% W/V PEG. Also, we did not identify any increased cleaved caspase 3, as an apoptotic cell death marker, in the whole cell population, at 8h after fusion (Suppl. Figure 2I). This indicates that PEG did not induce cell death under the applied conditions.

### Characterization of insulin+human/rat β-MSC heterokaryon cells revealed the expression of human as well as rat beta cell markers

**Figure 3:**
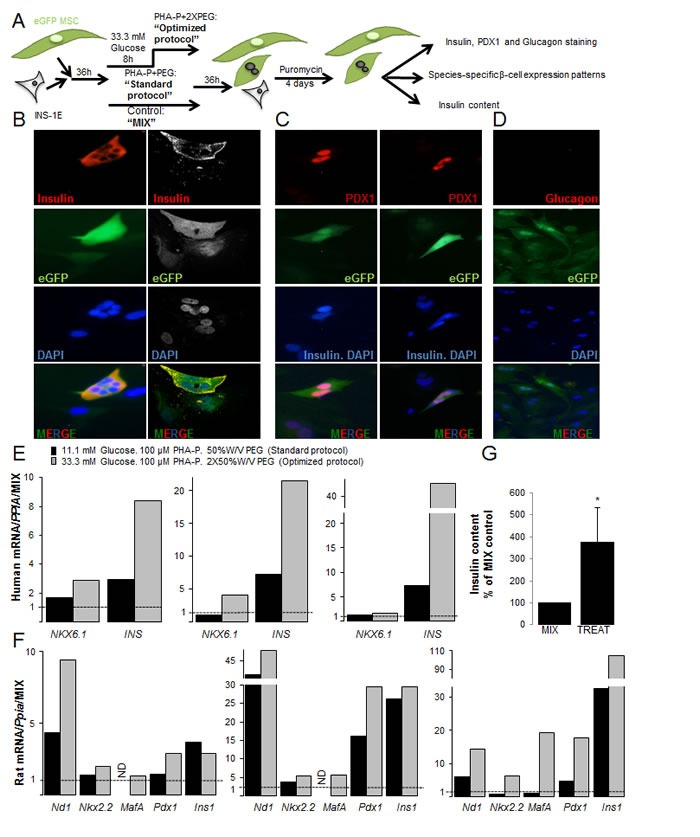
Characterization of insulin+human/rat β-MSC heterokaryon cells (**A**) Schematic illustration of the strategy for generation of fused human eGFP^+^MSCs/rat mCherry^+^INS-1E cells and their subsequent analysis. (B-G) eGFP^+^MSCs and mCherry^+^INS-1E cells were treated with PHA-P and PEG, 4 days after puromycin selection. (**B**) Triple labeling for insulin, eGFP and DAPI revealed polyploid insulin^+^eGFP^+^β-MSCs by fluorescence microscopy (left) or CLSM (right). (**C**) Triple staining for PDX1, DAPI and insulin shows PDX1^+^eGFP^+^insulin^−^ polyploid (left) as well as PDX1^+^eGFP^+^insulin^+^ polyploid cells (right; all 400X). (**D**) All cells were glucagon^−^, (200X). (E, F) RT-PCR analysis of human (**E**) and rat (**F**) specific mRNA sequences of β-cell markers shown from three independent experiments relative to mixed MSC/INS-1E cells and normalized to human (E) or rat Cyclophilin A (F). (**G**) Insulin content from mixed and treated cells under the optimized condition normalized to protein concentration and shown as percentage of mixed cells. All analyses are from at least three independent experiments from three different MSC donors shown in separate graphs (E, F) or as means ± SEM (G). ND = not detected. *P< 0.05 TREAT compared to MIX.

To further characterize the β-MSC heterokaryons, human eGFP^+^MSCs were mixed with INS-1E cells and treated with the standard or optimized protocol. Puromycin selection enabled isolating human MSCs and human/rat β-MSC heterokaryons (Figure 3A).

Although under the optimized 2XPEG fusion protocol we did not see improved fusion by elevated glucose, the combination of 8-hour pre-culture with 33.3 mM glucose and 10% FCS before fusion under the optimized protocol resulted in higher expression of *MAFA* and *insulin* in human MSC/islet cells (Suppl. Figure 3A) compared to 5.5 mM glucose (the basal glucose concentration for human beta cells) at 3% or 10% FCS. In order to apply the same pre-culture/fusion protocol to both MSC/INS-1E and MSC/human islets, we selected the glucose pre-culture with the optimized fusion protocol (MSC/β-cell ratio 1:11, 100 μg/ml PHA-P, 2X 50% W/V PEG) for all subsequent experiments as the best condition.

Our results showed eGFP and insulin co-positive heterokaryons (Figure 3B, Suppl. Figure 3B). Staining for PDX1 in red and insulin (cytoplasmic localization) and DAPI (nuclear localization) in blue showed quadruple-positive eGFP^+^DAPI^+^insulin^+^PDX1^+^ cells or triple positive eGFP^+^DAPI^+^insulin^−^PDX1^+^ (Figure 3C), but no eGFP^+^DAPI^+^insulin^+^PDX1^−^ cells. All heterokaryons were eGFP^+^glucagon^−^ (Figure 3D).

Next, we compared human and rat beta cell gene expression patterns [38] in the fused rat-human β-MSCs subjected to 100 μg/ml PHA-P/ 50% W/V PEG (standard protocol) or to 33.3 mM glucose pre-treatment/100 μg/ml PHA-P/ 2X 50% W/V PEG (optimized protocol). Data from three MSC donors are shown separately in order to see the inter-individual differences (Figure 3E, 3F, Suppl. Figure 3C, D). β-cell gene expression was not detected in eGFP-MSCs (not shown).

Higher expression of the human β-cell genes, *NKX6.1* and *insulin*, was observed under optimized compared to standard protocol conditions in all 3 experiments (Figure 3E). Higher mRNA levels of human *PAX4* were detected in two and *Neurogenin3 (NGN3)* in one isolation, while rat *Pax4* and *Ngn3* was not detected in any samples. This suggests the induction of early β-cell markers by human MSCs (Suppl. Figure 3C, D).

Rat *Nd1*, *MafA*, *Nkx6.1* and *Pdx1* as well as *Slc2a2*, *Ins1* and *Gck* mRNA** was increased under the optimized fusion protocol, suggesting that MSCs increased INS-1E-originated markers (Figure 3F, Suppl. Figure 3D; in two out of three experiments). Taken together, screening of β-cell gene expressions under the optimized fusion condition showed higher expression of β-cell markers compared to the standard protocol. The higher production level of insulin was confirmed by the increased insulin content (Figure 3G) in treated compared to mixed control.

### Characterization of insulin+ human β-MSCs

**Figure 4:**
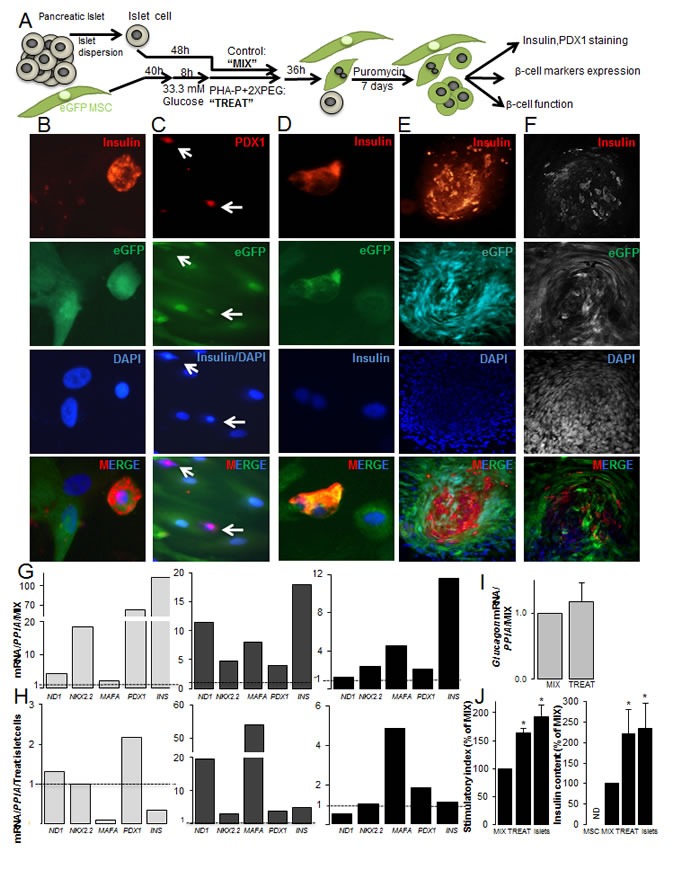
Fusion of human MSCs with human islet cells results in insulin+β-MSCs (**A**) Schematic illustration of the strategy for generation of fused human eGFP^+^MSCs/human dispersed islet cells. Mixed cells are treated with PHA-P and PEG (optimized protocol) and MSCs and β-MSCs selected by puromycin and analyzed subsequently. (**B**) Triple positivity for insulin, eGFP and DAPI in a β-MSC (400X). (**C**) PDX1, insulin and DAPI co-staining shows a PDX1^+^eGFP^+^insulin^−^ cell and a PDX1^+^eGFP^+^insulin^+^ cell (white arrows) (200X). (**D**) A human eGFP^+^insulin^+^β-MS heterokaryon (400X) (E, F) Insulin, eGFP, and DAPI triple positive cells in an islet like cluster (100X) by (**E**) fluorescence microscopy and (**F**) CLSM (G-I) The relative mRNA expression of human β-cell (**G**, **H**) and alpha-cell (**I**) markers normalized to (G, I) mixed (dotted line) MSC/dispersed islet cells or (H) mature islet cells (dotted line) from the same batch. RNA expression was normalized to human Cyclophilin A *(PPIA)*. (**J**) The stimulatory index denotes the amount of insulin secreted during 1h stimulation at 16.7 mM glucose divided by that of 1h at 2.8 mM glucose. Insulin content is from the same islet preparations; human dispersed islets, mixed and treated cells under the optimized condition normalized to protein concentrations. MSCs alone were added as –control. Data are shown as percentage of mixed cells. All analyses are from at least three independent experiments from three different islet and MSC donors shown in separate graphs (G, H) or as means ± SEM (I, J). *P< 0.05 TREAT compared to MIX.

To extend this fusion protocol to fully human β-MSCs, human eGFP^+^MSCs were cultured with dispersed human islet cells on the 804G matrix [39] with the same optimized fusion protocol (pre-treatment with 33.3 mM glucose and fusion with 100 μg/ml PHA-P/ 2X 50% W/V PEG). 36 hours later, cells were exposed to 10 μg/ml puromycin for 7 days which resulted in two main populations; MSCs and fused MSC/islet cells (Figure 4A).

Successful fusion was observed by double-positivity for insulin and eGFP (Figure 4B). Labeling of MSC/islet cells for eGFP, PDX1, insulin and DAPI showed quadruple-positive eGFP^+^DAPI^+^insulin^+^PDX1^+^ cells or triple positive eGFP^+^DAPI^+^insulin^−^PDX1^+^, but no eGFP^+^DAPI^+^insulin^+^PDX1^−^ cells (Figure 4C). These quadruple or triple positive cells appeared as synkaryons, which consisted of one single nucleus. We rarely observed insulin^+^eGFP^+^ polyploid cells (Figure 4D), which was in contrast to human/rat fused cells, where almost all insulin^+^eGFP^+^ were polyploid. Some of them formed dense islet like clusters (Figure 4E, 4F); such structures were observed in all human islet preparations from different donors. Insulin/eGFP double positive cells within the cluster were confirmed by confocal microscopy (Figure 4F). We also observed CD105^+^insulin^+^ cells in treated MSC/islet cell sections (Suppl. Figure 4C), while CD105 was rarely seen in control human islets and did not colocalize with insulin (Suppl. Figure 4A, B), which is in confirmation with previous reports [40, 41]. These densely formed islet like clusters made an accurate counting and even FACS analysis of the fused cells to exactly quantify fusion efficiency difficult.

We compared expression of human β-cell markers [38] under optimized and standard protocol conditions and observed higher induction of *ND1*, *NKX2.2*, *MAFA* and *INS* under the optimized fusion protocol in each of the 3 independent experiments (Suppl. Figure 4D). When human β-cell gene expression patterns in the treated conditions were compared with mixed controls, higher expression of the β-cell specific genes *NEUROD1*, *NKX2.2*, *MAFA*, *PDX1* and *insulin* (Figure 4G) was observed in all three independent experiments from different MSCs and islet donors. In order to compare β-cell specific gene expression to mature islets, mRNA of the 3 batches of mixed MSC/islet cells was also normalized to the respective mature control islet expression. Again, data from three MSC and three islet donors are shown separately and are fluctuating in their gene expression. Because of the inter-individual changes in the absolute gene expression as well as the stimulated conditions among the donors, data do not allow any further interpretation. Fused cells under the optimized conditions have a comparable amount of *PDX1* as well as *insulin* mRNA to mature islets, while *NEUROD1* and *MAFA* were increased in two out of three experiments (Figure 4H). To identify changes in alpha cell specific gene expression of fused cells, we analyzed glucagon levels, which were similar in both treated and mixed control conditions (Figure 4I). Functionality of fused cells was proven by glucose stimulated insulin secretion, which was 1.6-fold increased in the treated compared to the mix control population, also insulin content was 2.2-fold increased (Figure 4J). Functionality of treated fused cells was also compared to mature dispersed human islet cells, which was 1.9-fold increased in islets compared to mixed control. MSCs alone did not secrete insulin; neither under basal low glucose nor under stimulated conditions. Insulin content showed the same level as fused cells (Figure 4J).

## DISCUSSION

Recent investigations have revealed that MSCs have the potential to generate insulin-producing cells [19, 37, 42, 43]. Here, we developed a fusion protocol of human MSCs with rat INS-1E cells and human islet cells. One of the first cell fusion was reported by Sorieul and Ephrussi in 1961 in two constantly co–cultured mouse cell lines; after three months, 10% of the cells formed hybrids due to spontaneous fusion [44]. We also observed 1.6% spontaneous fusion of human MSCs and rat INS-1E cells already after 1 week of culture, which prompted us to foster such fusion by optimized protocols. Our approach resulted in β-MSC fused cells, which carried β-cell markers. Newly generated insulin-producing polyploid cells expressed nuclear PDX1 and cytoplasmic insulin. Better MSC-rat beta cell fusion efficiency was achieved by an optimized protocol of an increased number of β-cells in the mixture at a ratio of 1:11 MSC/INS-1E cells at PHA-P concentration of 100 ug/ml and a 2^nd^ addition of 50% W/V PEG to the cell mixture. This protocol resulted in 5-fold more heterokaryons as compared to the 1^st^ standard protocol.

Literature data show that the rate of fusion increases following tissue damage in order to restore the function of impaired tissue. For instance, the fusion of cardiomyocytes with mesenchymal stromal cells increases in low pH compared to high pH, suggesting the induction of mesenchymal-cardiomyocytes fusion event during ischemia *in vivo* [45]. Under conditions of elevated glucose, we also improved fusion efficiency. In addition, we observed higher levels of specific beta cell transcription factor expression in 33.3 mM compared to basal glucose levels in fused INS-1E as well as in fused human islet cells.

The improved MSC-rat beta cell fusion highly correlated with increased polyploidy, human β-cell marker expression as well as higher insulin content. In all experiments of fused MSC/rat INS-1E cells or MSC/human islet cells, increased fusion efficiency increased *PDX1* and *insulin* expression.

PDX1 is expressed as the first determination factor towards endocrine lineages. PDX1-expressing cells can differentiate into all pancreatic cell types; exocrine, endocrine and pancreatic ducts and thus serve as multipotent pancreatic progenitor cells [46]. Importantly, PDX1 positive cells have the capacity to proliferate [47]. The 2^nd^ important factor, which stimulates development into all islet cells, is NGN3 [48]. NGN3 drives expression of additional transcription factors such as PAX4, NEUROD1, NKX6.1, NKX2.2 and MAFA leading to the specific β-cell fate [49-51]. Adult β-cells express all of these factors except NGN3 and PAX4 [38].

NGN3 is only temporarily activated and often expressed at low levels. This could be one reason why we have only detected *NGN3* expression in one single experiment, where its expression was driven by MSCs, as specifically human and not rat *Ngn3* was detected in the optimized protocol. In the same experiment, also elevated *PAX4* expression was observed.

Fusion of rat/human islet cells with hMSCs using the optimized protocol induced human β-cell transcription factor expression *NKX6.1* and *MAFA*, which originated from the human MSCs. Additionally, β-cell transcription factors from rat β-cells (*Neurod1*, *Nkx2.2*, *MafA*, *Pdx1*) and genes related to β-cell function *(insulin*, *Glut2* and *Glucokinase)* were detected [51, 52].

The optimized protocol showed elevated levels of *NEUROD1*, *NKX2.2*, *MAFA* and *insulin* together with increased cellular insulin content also in fused human MSC with dispersed islet cells. The overall β-cell marker expression reached levels of mature islets confirming a human β-cell like phenotype of the fused MSC/islet cells.

In addition, β-cell transcription factor *PDX1* was higher in fused MSC/ dispersed islet cells than in mature islet cells. PDX1 plays a role in transition waves of developmental process as well as in mature β-cells as the promoter for glucose induced insulin transcription [51, 52]. The combination of markers for developmental factors together with those of mature beta cells suggest a mixed population of immature and mature beta-like cells. This hypothesis was strengthened by immunostaining of PDX1 and insulin, which also showed two cell populations: insulin^−^PDX1^+^ cells and insulin^+^PDX1^+^ cells, considered as immature and mature beta-like cells, respectively.

The difficulty in our study was the high variation of β-cell marker expression levels in the individual mixed/fused samples from different MSC and islet cells, which did not allow us to obtain a robust transcription marker analysis and conclusions on the cell differentiation state [53]. While human MSC/rat INS-1E cells showed polyploid heterokaryons, fused human MSC/dispersed islet cells showed no obvious polyploidy by immunostaining, these were rather synkaryons, suggesting the possibility of already fused nuclei and development of islet like cluster cells as hybrids. Such hypothesis needs further proof in long-term culture experiments. Their survival and potential glucose lowering effect in a diabetic environment, as suggested from a previous fusion study of rodent MSCs and islet cells [37] needs to be tested in further studies.

The high number of rat-human polyploid cells as stable interspecies heterokaryons provides a model for further investigation of epigenetic and genetic variation during cell differentiation to a β-cell phenotype. Our RT-PCRs showed induction of human beta cell markers in fused cells, which originated from both rat and human genes. This indicates reprogramming in fused cells, as shown before [37]. Here we did not check any changes in HLA types of the islet or MSC donors after fusion, but a transfer of the autologous HLA type from the MSC to the donor islets might be possible through fusion, such as observed before after cell fusion [54] and such as observed here with the human MSC genes to rat beta cells. Such ideas need further proof in the future.

In conclusion, we established a rapid and virus-free optimized fusion protocol of adherent MSCs and beta cells and show functional fused β-MSCs that express beta cell markers.

## MATERIALS AND METHODS

### Cell Culture

Human bone marrow cells from three healthy female donors were isolated at the University Hospital Hamburg-Eppendorf (UKE) and human mesenchymal stromal cells were isolated and purified as described previously [55]. Approval was granted by the UKE ethical committee. Concisely, bone marrow cells were cultured in AlphaMEM medium (Lonza, Basel, Switzerland) supplemented with 5% Platelet lysate [55], 10 I.U./mL heparin (Ratiopharm GmbH, Ulm, Germany) and 1% glutamax (Lonza, Basel, Switzerland). Medium was changed after two days and adherent cells were washed twice with phosphate-buffered saline (PBS) to omit other bone marrow cells. Then, growth medium was changed twice weekly. To identify colony forming unit fibroblasts (CFU-F), cells were plated in low density, fixed with methanol for 3 min and stained with 1% crystal violet for 5 min after 14 days. Confluent cells (passage 0) were seeded in new plastic adherent flasks till passage 3 and split to 1:3 [56]. Then, MSCs were stained for antigen surface marker expressions by flow cytometry or immunostaining. To confirm their capacity of a differentiation potential into mesodermal lineages, MSCs were induced to differentiate to adipocytes and osteoblasts as described [55]. Cell viability was assessed by Trypan blue (Invitrogen, Carlsbad, CA, USA) exclusion staining according to the manufacturer's protocol.

Human islets and the rat insulinoma cell line (INS-1E) [57] were cultured in their respective medium (CMRL (Invitrogen), RPMI1640 (GE Life Sciences/PAA Laboratories, Inc Little Chalfont, United Kingdom)) supplemented with 10% FCS, 1% L-glutamine and 100 U/ml penicillin/100mg/ml streptomycin (all GE Life Sciences/PAA Laboratories, Inc). INS-1E medium was supplemented with 50 μM β-mercaptoethanol (Merck) and 1 mM sodium pyruvate (GIBCO, Carlsbad, CA, USA) and 10 mM HEPES (Sigma-Aldrich). Human islets were isolated from 11 pancreases of non-diabetic organ donors at the University of Lille and Prodo Laboratories (Irvine, CA, USA) as described before [58] and cultured in suspension dishes for 48h. Informed consent was obtained from all subjects or their relatives. Islet purity was greater than 95% as judged by dithizone staining (if this degree of purity was not achieved by routine isolation, islets were handpicked). To disperse islets into single cells, accutase (Invitrogen) was added at 37°C for 15min and the cell suspension was mixed.

### Amplification of mCherry/eGFP LeGO virus, virus infection and stable cells

EGFP-puromycin and mCherry-zeocin plasmids were used as described previously (http://www.LentiGOVectors.de, [59]). To produce the lentiviruses, HEK 293T cells were co-transfected with cell marker drug-resistance vectors (eGFP-puromycin / mCherry-zeocin), Gag/ Pol (viral capsid), Rev (reverse transcriptase) and envelope plasmid via lipofectamine 2000 (Invitrogen) according to the manufacturer's instruction. After 12h incubation, medium was replaced with fresh medium, and supernatant was harvested 12h later. Virus was concentrated by centrifugation for two rounds at 50,000 xg for 2h. MSC or INS-1E cells were infected with eGFP-puromycin (green fluorescence) or mCherry-zeocin (red fluorescence) respectively.

MSC and INS-1E adherent cells were infected with multiplicity of infection (MOI) of 10/20 eGFP-puromycin /mCherry-zeocin lentiviruses respectively in the presence of 8 μg/ml Polybrene (Sigma-Aldrich). Plates were centrifuged at 1000 xg for 1h and cultured. After 24h, media was changed and 72h later, MSCs were treated with 0.5 to 2.5 μg/ml puromycin (Sigma-Aldrich) and INS-1E with 20 to 100 μg/ml zeocin (Invitrogen) antibiotics gradually to obtain eGFP^+^ or mCherry^+^ stable cells.

### Cell fusion

The cell fusion protocol was adapted from a previous standard protocol [60]. Phytohemagglutinin (PHA-P, Sigma-Aldrich) diluted in serum free RPMI1640 at 10 and 100 μg/ml, was added for 30 min and 37°C prewarmed-PEG (Sigma-Aldrich) was added dropwise in the dark at concentrations of 12.5, 25 and 50%W/V for 40s. PEG was diluted gradually by adding drop-by-drop serum free RPMI1640 medium to diminish the effect of osmotic pressure variations on cells. In some experiments, PEG was added a second time after 30 min (2XPEG).

Stable eGFP^+^MSCs were mixed with dispersed human islets and treated for 30 min with prewarmed-PEG at 37°C (Sigma-Aldrich), which was added dropwise in the dark at concentrations of 50%W/V for 50s. To select β-MSC heterokaryons and MSCs, non-fused INS-1E cells/human dispersed islets were eliminated at 36h after PHA-P/PEG treatment by adding 2.5 or 10 μM puromycin to the media for 4 or 7days respectively.

### Immunofluorescence

To evaluate the surface antigen expression, MSCs, eGFP^+^ MSCs or islets were Bouin-fixed, pelleted in 1% agarose and paraffin-embedded as described before [61]. After deparaffinization and high pH antigen retrieval solution (vector labs, CA, USA), MSC and eGFP^+^ MSC sections were stained with rabbit anti-CD105 or anti-CD90, mouse anti-CD73, rabbit anti-IgG or mouse anti-IgG1 (all Abcam, Cambridge, UK) followed by secondary Cy3 donkey anti-rabbit, Cy3 donkey anti-mouse or FITC donkey anti-mouse (all Jackson, ImmunoResearch Laboratories, West Grove, PA). Additionally, eGFP^+^ MSC sections were stained with rabbit anti-GFP and rabbit anti-IgG (all Abcam) followed by secondary FITC donkey anti-rabbit (Jackson). Islet sections were stained with guinea pig anti-insulin (DAKO, Hamburg, Germany) and rabbit anti-CD105 (Abcam) as well as Cy3 or FITC donkey anti-guinea pig and Cy3 or FITC donkey anti-rabbit (all Jackson).

For characterization of mCherry^+^ INS-1Es and puromycin selected β-MSCs, cells were fixed with 4% paraformaldehyde in PBS for 30 min and permeabilized by 0.5% Triton X100 for 4 min at room temperature. Immunostaining with guinea pig anti-insulin (DAKO), rabbit anti-PDX1 (Abcam) or rabbit anti-glucagon antibody (DAKO) and Cy3, FITC or AMCA donkey anti-guinea pig as well as Cy3 donkey anti-rabbit (all Jackson) was performed as described previously [61]. Nuclei were visualized with 6-diamino-2-phenylindole (DAPI) (Vector labs). Fluorescently stained cells were analyzed with a Nikon MEA53200 microscope (Nikon GmbH, Düsseldorf, Germany) /Zeiss confocal laser scanning microscope (CLSM) 780 with ELYRA PS.1 (Zeiss, Oberkochen, Germany), and images were recorded using NIS-Elements software (Nikon)/ ZEN2011black edition (Zeiss).

### Glucose stimulated insulin secretion (GSIS) and Insulin content

An equal number of islet cells, mixed or treated islet-MSC cells, INS1-E and mCherry INS1-E cells were used to perform glucose-stimulated insulin secretion as described before [61]. Cells were pre-incubated in Krebs-Ringer bicarbonate buffer (KRB) including 2.8 mM glucose for 30 min which was replaced by 2.8 mM glucose KRB for 1 h (basal), followed by an additional 1 h incubation in 16.7 mM glucose KRB (stimulated insulin secretion). Cells were lysed in lysis buffer (20 mM Tris acetate, 0.27 M sucrose, 1 mM EDTA, 1 mM EGTA, 50 mM NaF, 1% Triton X-100, 5 mM sodium pyrophosphate, 10 mM β-glycerophosphate plus protease and phosphatase inhibitors; Pierce) and secreted insulin and insulin content from lysed measured by mouse (for rat INS-1E cells; cross reactivity is 100%) or human insulin ELISA kit (for human islets; ALPCO Diagnostics, Salem, NH, USA). Insulin content was normalized to total protein measured by BCA protein assay kit (Pierce, Rockford, IL, USA) as described before [62].

### Quantitative RT-PCR analysis

After puromycin selection, total RNA was extracted with TRIzol (Invitrogen) according to the manufacturer's protocol and RT-PCR performed as described previously [63]. Rat and human-specific primers were designed using vector NTI advanced ™ 11 Software (Invitrogen) and RT-PCR performed with SYBR Green 2X PCR Master Mix (Applied Biosystems, Darmstadt, Germany). Primers used were 5^/^ AAACGGTTCCTTAGGGCAAT3^/^/ 5^/^CAGTCTCACTGCCCCAACTT3^/^ (rat *Taf1*) and 5^/^CCTGTCCCCTCAGTTCATGT3^/^/ 5^/^CCCATGGTCTTCTTCTGCAT3^/^ (*mCherry*). TaqMan® Real-time RT-PCR was performed using the 2X TaqMan Universal PCR Master Mix with an ABI StepOne Plus Cycler (Applied Biosystems). Reactions were performed in technical duplicates in a volume of 10 μl with specific primers and probes. TaqMan® primers used were of *PPIA* (Hs99999904_m1), *INS* (Hs02741908_m1), *PDX1* (Hs00426216_m1), *NEUROG3* (Hs01875204_s1), *NKX6.1* (Hs00232355_m1), *NKX2.2* (Hs00159616_m1), *NEUROD1* (Hs01922995_s1), *MAFA* (Hs01651425_s1), *PAX4* (Hs00173014_m1), *Ppia* (Rn00690933_m1), Ins1 (Rn02121433_g1), *Pdx1* (Rn00755591_m1), *Slc2a2* (Rn00563565_m1), *Gck* (Rn00688285_m1), *Neurog3* (Rn00572583_s1), *Nkx6.1* (Rn1450076_m1), *Nkx2.2* (Rn04244749_m1), *Neurod1* (Rn00824571_s1), *MafA* (Rn00845206_s1), *Pax4* (Rn00582529_m1) for human/ rat r(Applied Biosystems). Results were calculated with the ΔΔC_T_ method. All gene expression data sets were normalized to the corresponding puromycin selected non-treated mixed cells or treated dispersed islets and normalized to housekeeping genes human *PPIA* or rat *Ppia*/*Taf1*. Samples were analyzed in duplicate for each transcript. In MSC/INS-1E experiments, it was not possible to measure the target gene in each control mix condition; therefore, we normalized to one single randomly chosen sample. Control experiments were performed with human islet and rat INS-1E cells to confirm rat or human primer specificity.

### Fluorescence-activated cell analysis (FACS)

MSCs were trypsinized, washed with PBS and 10^5^ cells in each condition were incubated with FITC conjugated anti-CD105, FITC conjugated anti-CD90 and PE conjugated anti-CD73 (positive markers) as well as FITC conjugated anti-MHCII, FITC conjugated anti-CD45, PE conjugated anti-CD34 (negative markers). FITC conjugated anti-IgG1, FITC conjugated anti-IgG2a and PE conjugated anti-IgG1 were used as negative isotype control (all Becton Dickinson Biosciences, Franklin Lakes, New Jersey, USA). 72h after LeGO Virus infection, the number of eGFP positive MSCs was quantified using a 488nm laser and 530/30 bandpass filter by FACS analysis.

For each analysis, we selected a minimum of 5000 cells for counting per condition. To quantify the number of rat/human β-MSC interspecies heterokaryons, live cells in different conditions plus non-infected control MSCs, INS-1E and human islet cells were trypsinized and evaluated by eGFP/mCherry double color on LSRFortessa (Becton Dickinson Biosciences) using 488nm laser and 530/30 bandpass filter (eGFP) and 561nm laser and 610/20 bandpass filter (mCherry) respectively.

For polyploidy FACS, cells were fixed by adding cold 70% ethanol (10^6^ cells/ml) dropwise and kept at 4°C overnight. Cells were washed two times with PBS and incubated with 2 μg/ml DAPI plus 5 μg/ml RNase in PBS solution for 30min at 37°C in the dark. Cells were measured with an UV laser (450/50 bandpass filter). The numbers of cells in different samples were counted by LSRFortessa (Becton Dickinson Biosciences). Data were analyzed by BD FACSDiva™ software 6.0 or Cyflogic 1.21.

### Western blot analysis

Cells were washed with PBS, lysed in lysis buffer (see above) and protein concentration determined with the BCA protein assay (Pierce). In all conditions, the same amount of protein was run on a NuPAGE 4-12% Bis-Tris gel (Invitrogen) and electrically transferred onto PVDF membranes. After 1h blocking at RT, PVDF membranes were incubated with rabbit anti-Cleaved Caspase 3, rabbit anti-tubulin or rabbit anti-GAPDH (all Cell Signaling Technology) overnight at 4°C, followed by horseradish-peroxidase-linked anti-rabbit IgG (Jackson). PVDF Membranes was developed by chemiluminescence assay system (Pierce) and analyzed by DocIT®LS image acquisition 6.6a (UVP BioImaging Systems, Upland, CA, USA). For +control, INS-1E cells exposed to 12h palmitic acid were used as described before [61].

### Statistical analysis

Data evaluation was done in a blinded manner by a single investigator (ZA) and statistical analyses were performed from at least three independent experiments from three individual human MSC batches and three different human islet organ donors. Values are presented as means ± SEM. Data were analyzed by two-sample one-tailed student's t-tests with equal variance. Significance was set at p<0.05.

## SUPPLEMENTARY MATERIALS FIGURES






