# Supplementary material for: β-MSCs: successful fusion of MSCs with β-cells results in a β-cell like phenotype
Source: Oncotarget. 2016 Jun 21;7(31):48963–77. doi: 10.18632/oncotarget.10214 (PMC5226484; doi:10.18632/oncotarget.10214)
Supplement: Supplementary file 1 [file oncotarget-07-48963-s001.pdf]

β-MSCs: successful fusion of MSCs with β-cells results in a beta-cell like phenotype

Supplementary Material

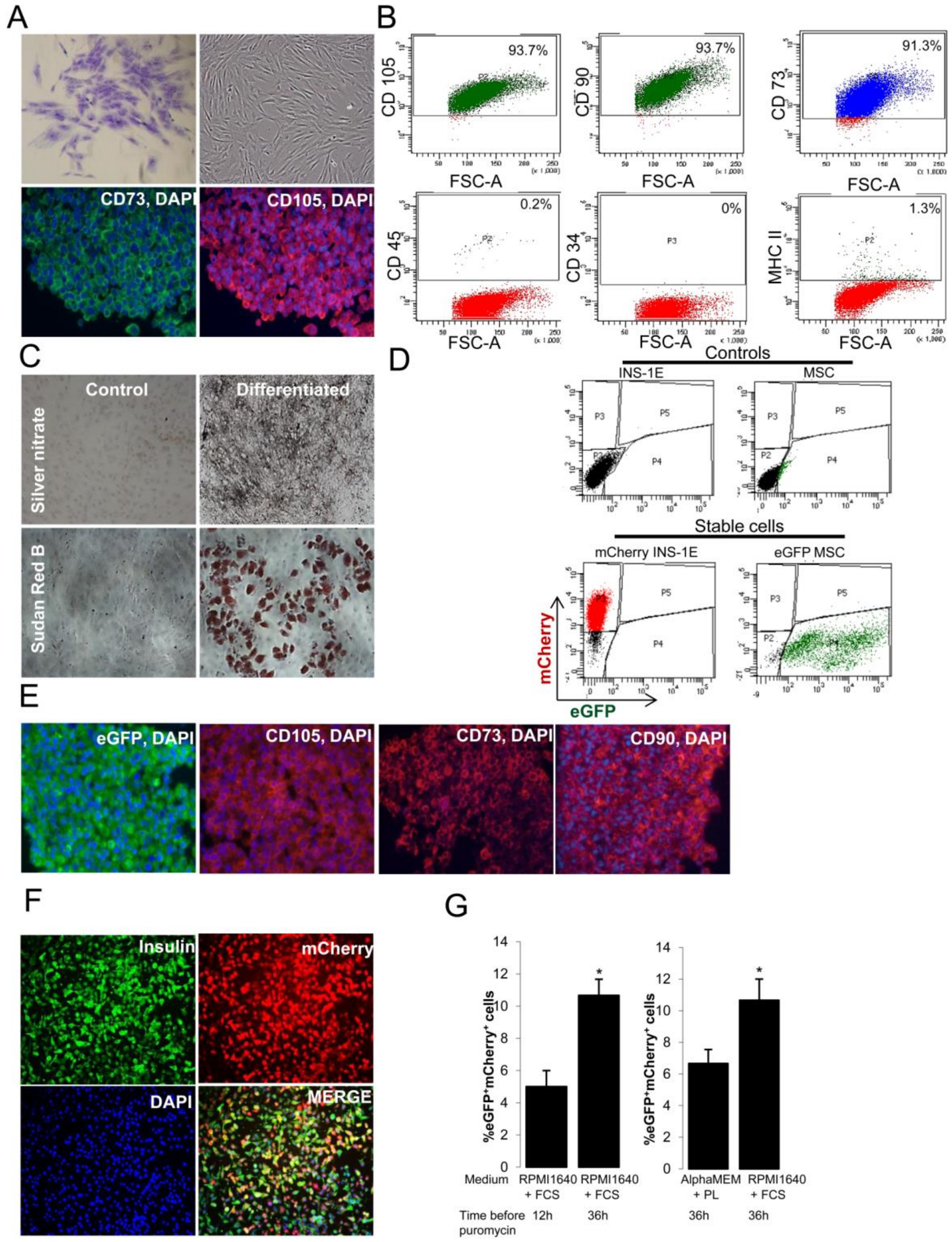

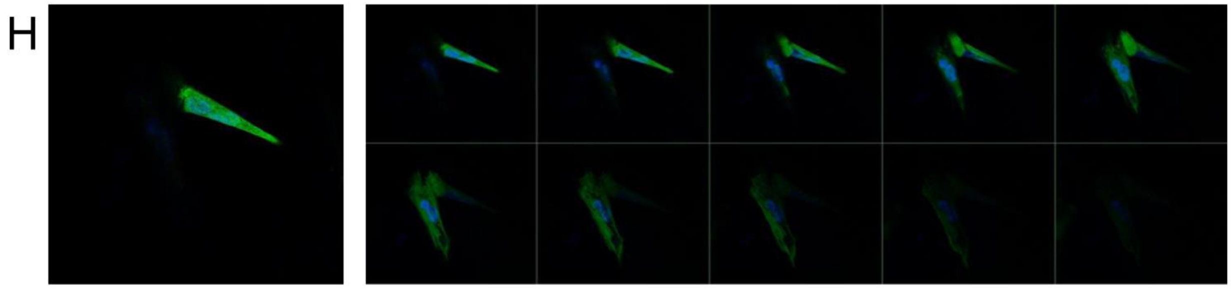

Suppl.Fig.1. Characterization of human bone marrow MSCs.

(A) Colony Forming Unit-Fibroblast (CFU-F) stained with 1% crystal violet as a marker of MSCs in culture after 1 week (left) and the shape of MSCs in long-term culture of around 3 weeks (right; 40X) as well as CD73 and CD105 staining of paraffin embedded MSC sections (200X). **(B)** Positive (CD105, CD90 and CD73; top) as well as negative (CD45, CD34 and MHC-II; bottom) MSC markers by flow cytometry **(C)** MSC differentiation potential after stimulation (see material and methods) into adipocytes and osteoblasts analyzed by Sudan red B and Silver nitrate, respectively (40X). **(D)** Efficiency of eGFP^{+}MSC and mCherry^{+}INS-1E stable cells shown by flow cytometry. **E)** EGFP, CD105 and CD73 and CD90 staining of paraffin embedded eGFP^{+}MSC sections (200X). **(F)** Insulin staining of mCherry^{+} INS-1Es (200X). **(G)** To select the optimal time point of puromycin, MSC/INS-1E fused cells were treated with puromycin after 12h or 36h. To select the optimum medium, MSC/INS-1E cells were maintained in AlphaMEM and platelet lysate (PL; MSC medium) or RPMI1640 and FCS (INS-1E medium) immediately after standard fusion protocol treatment for 36h. The percentage of eGFP^{+}/mCherry^{+} cells was counted under the fluorescent microscope. (H) Z-stack movie (left) and serial sections (right) from eGFP^{+}MSC homokaryons (0.4 µm) (400X). *P< 0.05 at RPMI1640 to AlphaMEM or 12h RPMI1640.

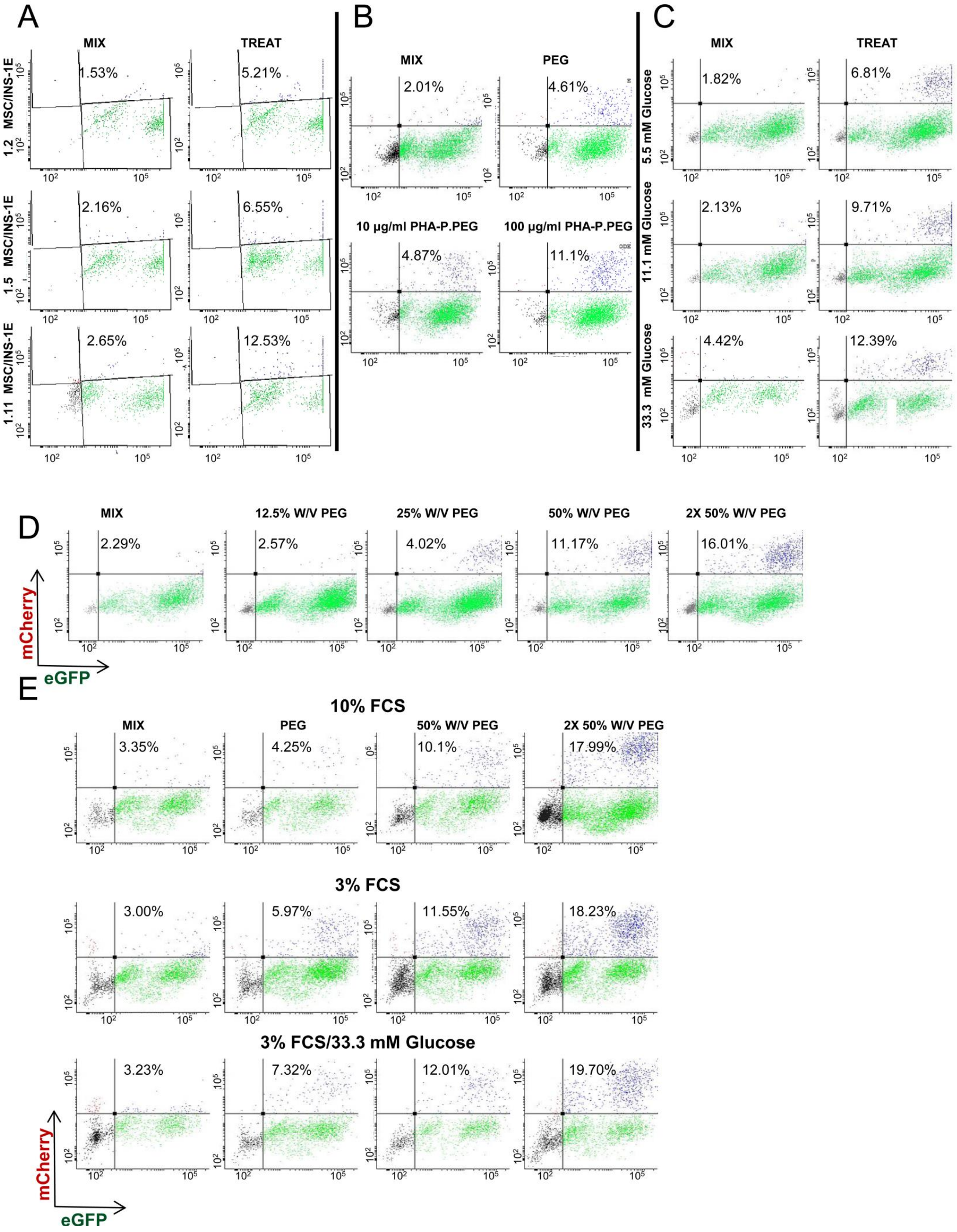
A
MIX
TREAT
1.2 MSC/INS-1E
1.53%
5.21%
1.5 MSC/INS-1E
2.16%
6.55%
1.11 MSC/INS-1E
2.65%
12.53%
B
MIX
PEG
2.01%
4.61%
10 µg/ml PHA-P.PEG
100 µg/ml PHA-P.PEG
4.87%
11.1%
C
MIX
TREAT
5.5 mM Glucose
1.82%
6.81%
11.1 mM Glucose
2.13%
9.71%
33.3 mM Glucose
4.42%
12.39%
D
MIX
12.5% W/V PEG
25% W/V PEG
50% W/V PEG
2X 50% W/V PEG
2.29%
2.57%
4.02%
11.17%
16.01%
mCherry
eGFP
E
10% FCS
MIX
PEG
50% W/V PEG
2X 50% W/V PEG
3.35%
4.25%
10.1%
17.99%
3% FCS
3.00%
5.97%
11.55%
18.23%
3% FCS/33.3 mM Glucose
3.23%
7.32%
12.01%
19.70%
mCherry
eGFP

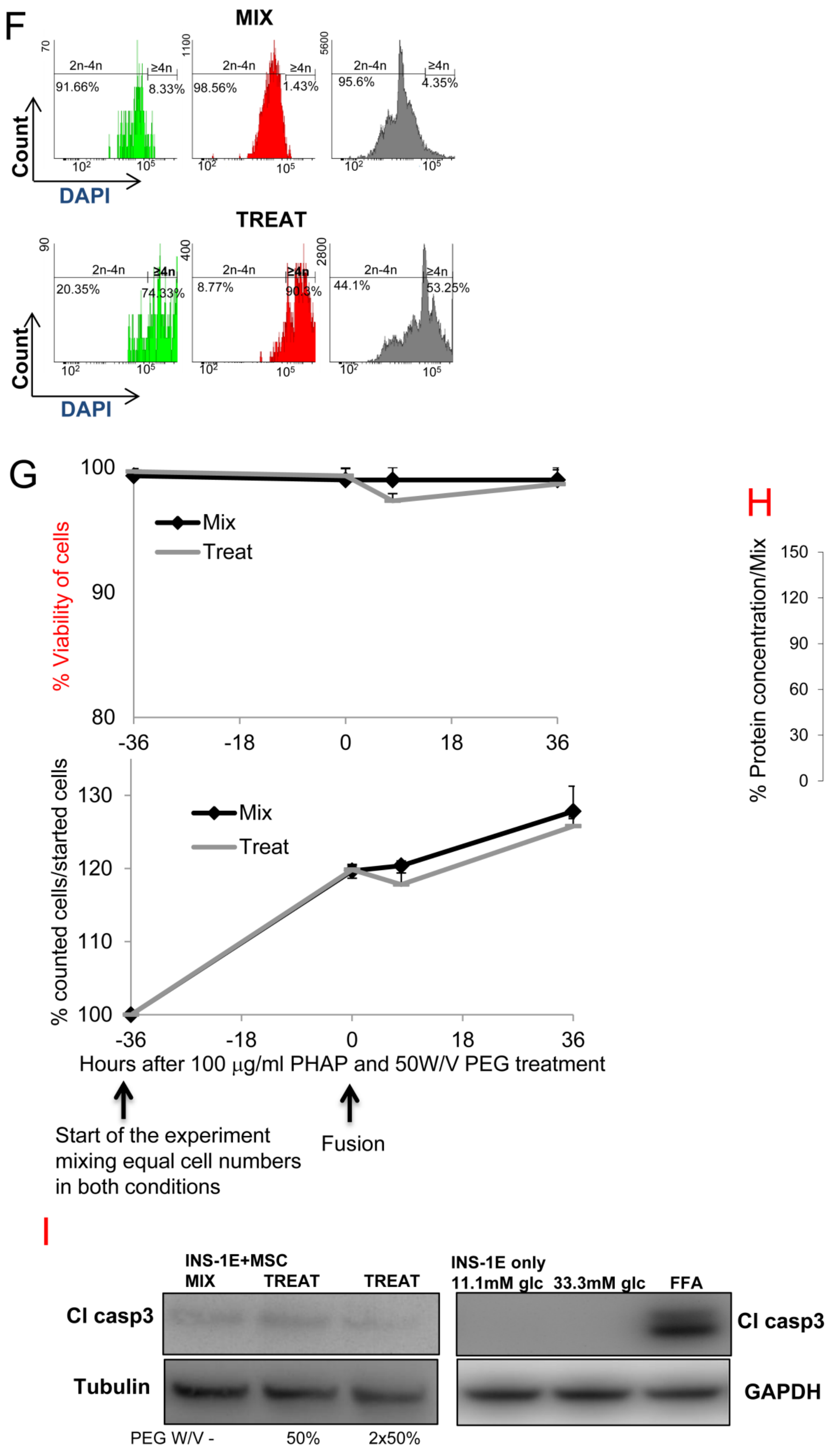
F
MIX
2n-4n
≥4n
91.66%
8.33%
98.56%
1.43%
95.6%
4.35%
Count
DAPI
TREAT
20.35%
74.33%
8.77%
90.3%
44.1%
53.25%
G
% Viability of cells
Mix
Treat
% counted cells/started cells
Hours after 100 μg/ml PHAP and 50W/V PEG treatment
Start of the experiment mixing equal cell numbers in both conditions
Fusion
H
% Protein concentration/Mix
MIX
TREAT
I
INS-1E+MSC
MIX
TREAT
TREAT
Cl casp3
Tubulin
PEG W/V -
50%
2x50%
INS-1E only
11.1mM glc
33.3mM glc
FFA
Cl casp3
GAPDH

Suppl.Fig.2. Optimization of cell fusion protocol.

(A-E) Representative scatter plots corresponding to the bar graphs in Fig.2A-E. **(F)** Representative histograms corresponding to the bar graphs in Fig.2G. Gating was set for control non-stained cells. (G) Percentage of trypan blue negative cells (upper panel) and cell numbers (lower panel) were counted during the experiment before and after fusion at 8h after PEG treatment. A representative field of 100 cells were counted for each treatment condition in triplicate (upper panel). Starting cell numbers were 55,000 eGFP+MSCs and 550,000 mCherry+INS-1Es. (H) Protein content in mix and treated cells at 8h after PEG treatment (optimized protocol). (I) Western blot analysis of cleaved Caspase 3 and Tubulin/GAPDH (loading controls) of treated (optimized fusion protocol with 2x50%PEG and fusion protocol with 2x50%PEG and mixed INS-1E/MSC) and of INS-1E cells only cultured for 12h at 11.1 mM glucose (-control), 33.3 mM glucose or 5.5 mM palmitate (+control). Blots are representative of 4 experiments.

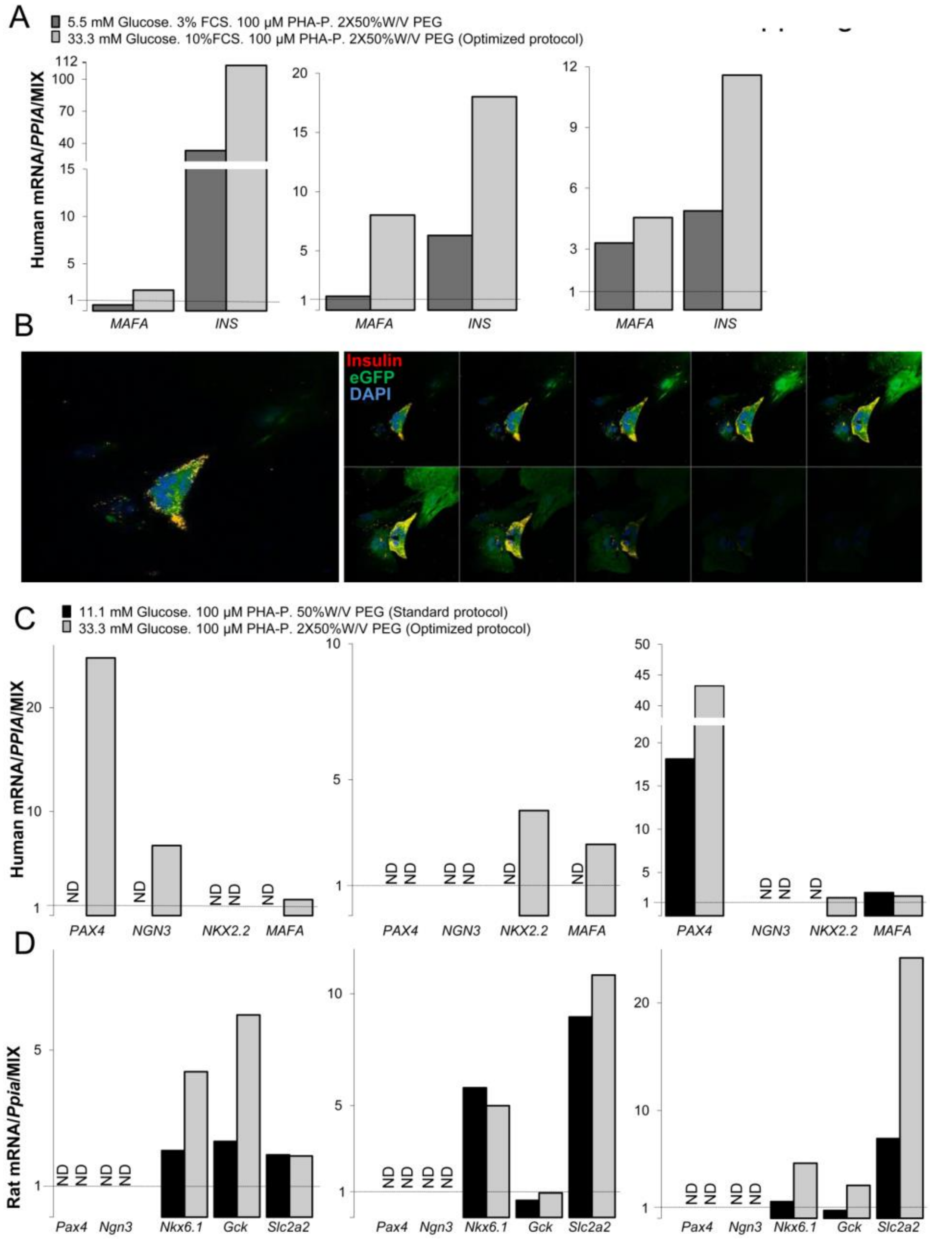

Suppl.Fig.3: Characterization of insulin⁺ human/rat β-MSC heterokaryon cells.

(A) The relative mRNA expression of *MAFA* and *Insulin* of treated normalized to mixed human MSC/dispersed islet cells and to human Cyclophilin A. **(B)** Z-stack movie (left) and serial sections (right) from a $eGFP^+insulin^+$ heterokaryon (0.4 µm) from $eGFP^+MSC/mCherry^+INS$-1E cells under the optimized fusion protocol. (400X) **(C,D)** Relative mRNA expression of human **(C)** as well as rat beta-cell markers **(D)** from treated $eGFP^+MSC/mCherry^+INS$-1E cells normalized to mixed control conditions. RT-PCR was normalized to human **(C)** or rat Cyclophilin A **(D)**. Results represent three independent experiments. All analyses are from at least three independent experiments from three MSC donors shown in separate graphs.

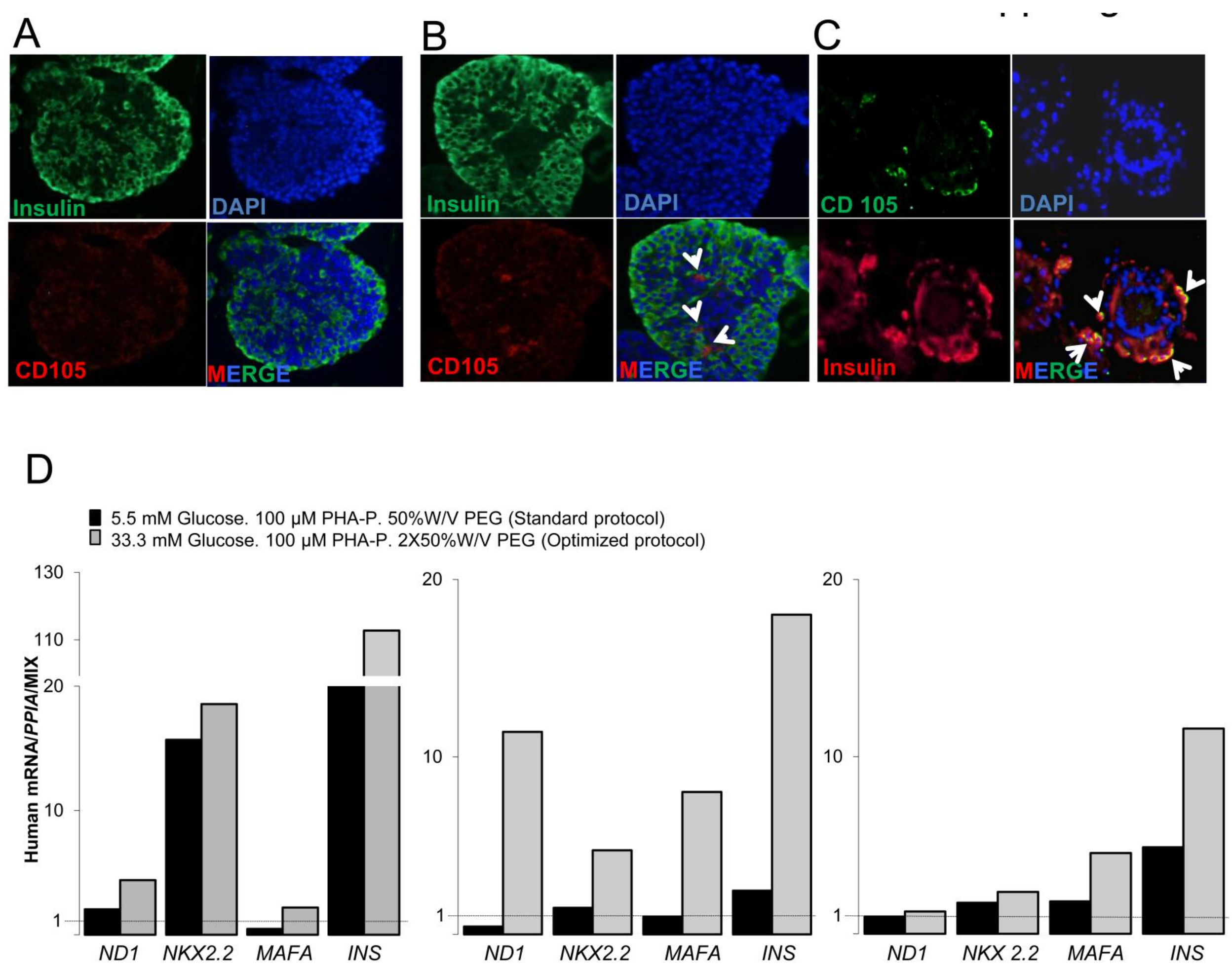

Suppl.Fig.4. Characterization of insulin^{+} human β-MSCs.

(A-B) Co-staining of insulin and CD105 in paraffin sections and fluorescent microscopical analysis **(A)** in a human islet with no CD105^{+} cells, **(B)** in a human islet with CD105^{+}insulin^{-} cells and (**C**) in fused MSCs with human islet cells with CD105^{+}insulin^{+} double positive cells. White arrows show CD105^{+} (**B**) and CD105^{+}insulin^{+} double positive cells (**C**; 300X). **(D)** The relative mRNA expression of human β-cell markers of treated human β-MSC cultures normalized to mixed MSC/dispersed islet cells as control and to human Cyclophilin A. The bar graphs represent three independent experiments from three different MSC and islet donors shown in separate graphs.
